# Theranostic Phthalocyanine and Naphthalocyanine Nanoparticles for Photoacoustic Imaging and Photothermal Therapy of Tumors

**DOI:** 10.7150/ntno.88892

**Published:** 2024-01-01

**Authors:** Yiran Tian, Nicole Carrillo-Malani, Kailin Feng, Joann Miller, Theresa M. Busch, Karthik M. Sundaram, Zhiliang Cheng, Ahmad Amirshaghaghi, Andrew Tsourkas

**Affiliations:** 1 Department of Bioengineering, University of Pennsylvania, Philadelphia, PA 19104, USA.; 2 Department of Radiation Oncology, Perelman School of Medicine, University of Pennsylvania, Philadelphia, PA, 19104, USA.; 3 Department of Radiology, Hospital of University of Pennsylvania, Philadelphia, PA 19104, USA.

**Keywords:** phthalocyanine, naphthalocyanine, photothermal therapy, photoacoustic imaging, theranostic

## Abstract

**Background:** Phthalocyanine (PC) and naphthalocyanine (NC) dyes have long garnered interest as theranostic agents for optical imaging and phototherapy due to their near-infrared absorbance, photostability, imaging contrast, and proven safety in clinical trials. Yet, only a small fraction of these dyes has been evaluated as photothermal therapy (PTT) agents for cancer treatment.

**Methods:** Nearly 40 distinct NC and PC dyes were encapsulated within polymeric PEG-PCL micelles via oil-in-water emulsions. The optimal NC/PC-loaded micelle formulations for PTT and photoacoustic (PA) imaging were identified through *in vivo* and *in vitro* studies.

**Results:** The most promising candidate, CuNC(Octa)-loaded micelles, demonstrated a strong PA signal with a peak absorbance at ~870 nm, high photothermal efficiency, and photostability. The CuNC(Octa)-loaded micelles exhibited heat generation as good or better than gold nanorods/nanoshells and >10-fold higher photoacoustic signals. Micelle preparation was reproducible/scalable, and the CuNC(Octa)-loaded micelles are highly stable under physiological conditions. The CuNC(Octa)-loaded micelles localize within tumors via enhanced permeability and retention and are readily detectable by PA imaging. In a syngeneic murine tumor model of triple-negative breast cancer, CuNC(Octa)-loaded micelles demonstrate efficient heat generation with PTT, leading to the complete eradication of tumors.

**Conclusions:** CuNC(Octa)-loaded micelles represent a promising theranostic agent for PA imaging and PTT. The ability to utilize conventional ultrasound in combination with PA imaging enables the simultaneous acquisition of information about tumor morphology and micelle accumulation. PTT with CuNC(Octa)-loaded micelles can lead to the complete eradication of highly invasive tumors.

## Introduction

The development of dual therapy and diagnostic imaging (theranostic) agents represents a rapidly growing field for the imaging and treatment of many types of solid malignancies, including melanoma, glioblastoma, and prostate cancer 1-4. Theranostic agents allow a treatment modality (e.g., radiation, thermal-ablation, high-intensity ultrasound) to be combined with a complementary imaging modality (e.g., PET, SPECT, MRI, optical imaging, or ultrasound) in a single disease therapy and localization platform 5-7. Amongst the many possible combinations of imaging and therapeutic modalities, the use of optical imaging with phototherapy offers a favorable option for the real-time imaging and treatment of cancer.

Phototherapy, which includes photodynamic therapy (PDT) and photothermal therapy (PTT), is being explored clinically as a treatment option for various forms of cancer 8, 9. PDT uses photosensitizers to produce singlet oxygen upon the administration of light 10, while PTT uses photothermal agents to enhance the laser-induced heating of cells and tissues 11. While PDT has perhaps garnered more attention, particularly in clinical trials, PTT may offer several important advantages. For example, unlike PDT, PTT is not sensitive to oxygen, which is often limited in the tumor microenvironment. Moreover, PTT does not require patients to remain in the dark to avoid skin toxicity.

Various dyes and nanoparticles have been explored as PTT agents, but gold nanoparticles and the small-molecule dye, indocyanine green (ICG), have perhaps received the most attention and remain the only two compounds to be tested in clinical trials to date 12-15. In a recent study, gold-silica nanoshells were administered intravenously to patients with prostate cancer. Tumors were successfully ablated in 94% of patients with no serious complications and no significant change in Prostate Symptom or Sexual Health scores 16. Although the results are promising, a significant concern with intravenously administering gold nanoparticles is their protracted elimination 17, 18. The long-term retention of ICG is less of a concern due to its small size and more rapid clearance; however, when used as a PTT agent for the treatment of metastatic breast cancer, the objective response rate was only 62.5%. The low efficacy is possibly due to poor distribution and selectivity following local administration, rapid clearance of dye from the injection site due to the small size of ICG, and/or thermal degradation during irradiation 19-23.

Phthalocyanines (PC) and Naphthalocyanine (NC) dyes could represent a favorable alternative to gold nanoparticles and ICG as a PTT agent. NC and PC are small-molecule organic dyes; many of these dyes and derivatives absorb in the near-infrared wavelengths, have high extinction coefficients (>10^5^ L mol^-1^cm^-1^), and high photostability 24. PC dyes are being evaluated in several clinical trials as photodynamic therapy (PDT) agents (NCT03769506; NCT01800838) and are generally considered safe, with some PC dyes having received clinical approval in other countries 25. Although NC/PC dyes have also been evaluated as PTT agents 26, 27, (or combined PDT and PTT) 28, the PTT properties of many NC/PC variants remain unexplored.

In addition to PTT, NC/PC dyes have also been found to generate excellent photoacoustic (PA) contrast 29-35. Co-registered photoacoustic (PA) and ultrasound (US) imaging represent a real-time and affordable technique for imaging disease 36, 37. The PA signal can be visualized in milliseconds, which is advantageous over other emerging imaging techniques, such as Raman spectroscopy and Optical Coherence Tomography, which generally require much longer acquisition times 38, 39. Also, in comparison to fluorescence imaging, PA imaging has improved sensitivity for tumors deeper than 2 mm and the ability to image deeper-seeded tumors (5-6 cm vs. 1 cm) while maintaining high spatial resolution (~100 µm) 40-43. PA imaging may allow nanoparticle accumulation to be monitored at a disease site, which can help guide PTT treatment. PA imaging may also enable satellite lesions to be identified 44, 45.

In this work, we screened nearly 40 distinct NC and PC dyes and derivatives after encapsulation within micelles to identify optimal formulations for PTT and PA imaging. The most promising candidate, CuNC(Octa)-loaded micelles, demonstrated a strong PA signal with a peak absorbance at ~870 nm, high photothermal efficiency, and photostability. The CuNC(Octa)-loaded micelles localize within tumors via enhanced permeability and retention and are readily detectable by PA imaging. In a syngeneic murine tumor model of triple-negative breast cancer, CuNC(Octa)-loaded micelles demonstrated efficient heat generation with PTT and led to complete ablation of tumors. Given these results, we envision that CuNC(Octa)-loaded micelles (and other additional candidates) will offer a powerful theranostic agent for PA imaging and PTT for real-time, image-guided therapy of localized solid malignancies.

## Results and Discussion

### Preparation and Characterization of NC and PC Micelles

**Figure 1:**
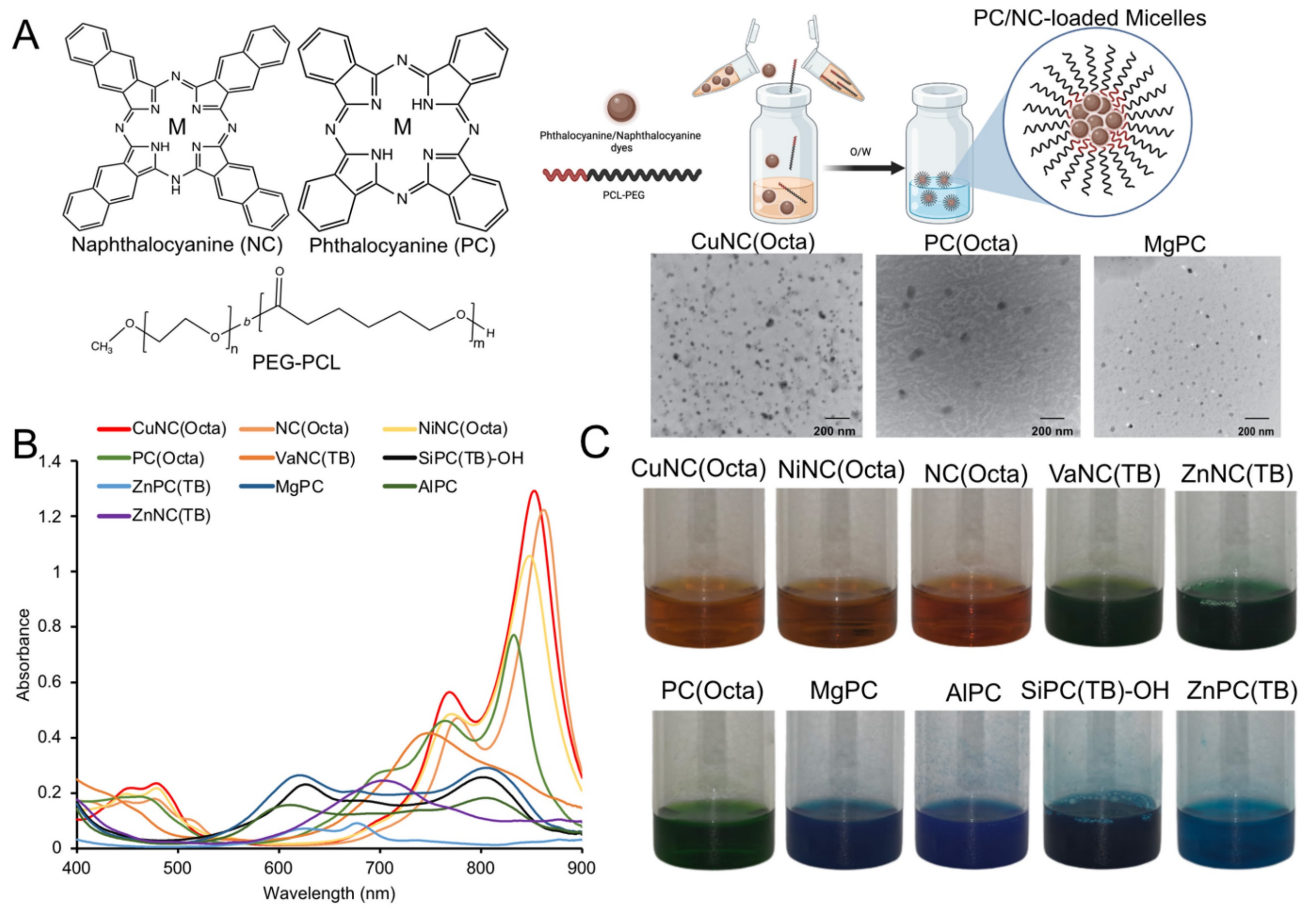
(A) Illustration of NC/PC-loaded micelles. Chemical structure of NC/PC and the solubilizing hydrophobic PCs and NCs within PEG-PCL micelles via oil-in-water (O/W) emulsions. Inset: TEM image of select NC/PC-loaded micelles (scale bar: 200 nm) (B) Raw absorbance spectrum of 10 different NC/PCs at 10 μM (based on the dye concentration). (C) The photographs of 10 different NC/PC-loaded micelle samples in water.

Twenty-eight phthalocyanine and nine naphthalocyanine dyes (Figure S1) were dissolved in organic solvent and loaded into polymeric micelles, composed of the amphiphilic diblock copolymer polyethylene glycol-polycaprolactone (PEG-PCL), via oil-in-water emulsions (Figure 1A). The hydrodynamic diameters of the micelle formulations ranged from 35 to 110 nm as measured by dynamic light scattering. Transmission electron microscopy images of select PC/NC micelles showed successful micelle assembly, which could be detected by the metal centers of the PC/NC dyes encapsulated within the hydrophobic core (Figure 1A). Micelles were mostly found to be negatively charged, which is consistent with the presence of a dense PEG shell (Table S1), although there were some notable exceptions. For example, SiPC-(TB)-OH-loaded micelles had a near neutral charge (-0.7 mV), and ZnPC(TB) micelles exhibited a slightly positive charge (4 mV), as we previously reported 46. For each PC/NC-loaded micelle, there was minimal batch-to-batch variability in the hydrodynamic diameter, polydispersity index (PDI), encapsulation efficiency, and zeta potential. The peak absorbances of the PC/NC-loaded micelles (Figure 1B, Figure S2) resided between 400nm and 900nm. Representative photographs of PC/NC-loaded micelle samples are shown in Figure 1C. All 37 micelle formulations were translucent and brown, green, or blue in color. The micelles were stable in solution, with no detectable signs of aggregation or precipitation and no significant change in the hydrodynamic diameter for at least 6 months. The CuNC(Octa)-loaded micelles were also stable in 10% fetal bovine serum (FBS) for at least 10 days, with no signs of aggregation or precipitation (Table S2).

### *In Vitro* Heating of NC and PC Micelles

**Figure 2:**
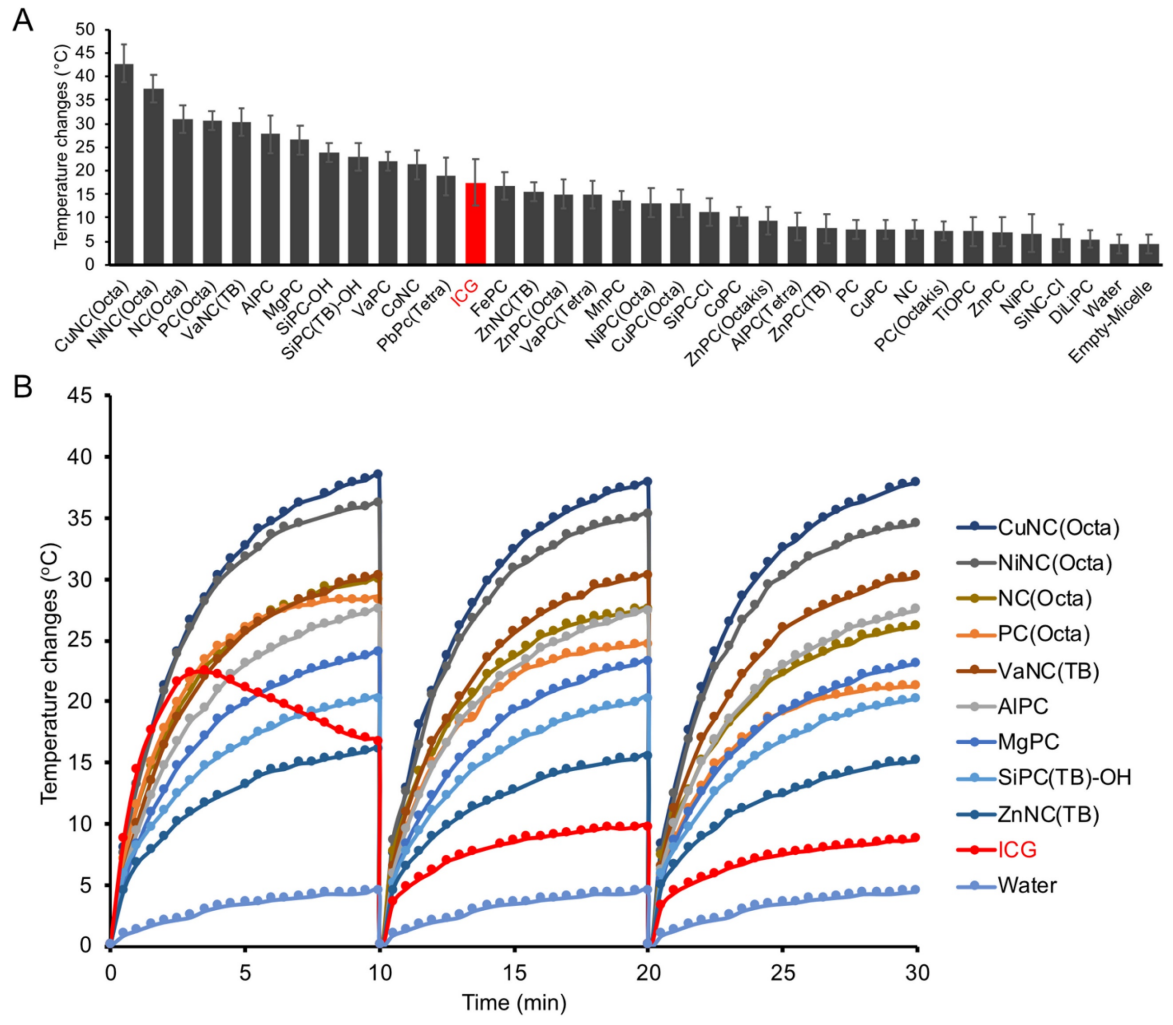
(A) Nearly 40 different NC/PC micelles were prepared, and 33 were irradiated with 808nm light (0.7 W/cm^2^). The temperature was measured after 10min. Micelles are plotted from those that generated the highest to lowest change in temperature at 10 μM (based on the dye concentration) and compared with ICG (red bar). (B) Select micelle samples and ICG were subject to cyclic heating/cooling. All NC/PC micelles exhibited excellent photostability, while ICG exhibited significantly less heating efficiency with time.

Each micelle formulation at a fixed dye concentration (10 µM) was evaluated in its ability to generate heat upon irradiation with an 808 nm laser (0.7 W/cm^2^) (Figure 2A). The temperature was recorded over 10 minutes with a fiber optic thermometer (Nomad, Qualitrol-Neoptix, Fairport, NY). Most NCs and PCs were found to generate a significant increase in temperature upon irradiation, with 12 formulations reaching a higher final temperature after 10 minutes than free ICG. We also attempted to load ICG into micelles for a fair comparison, but the amphiphilic nature of ICG prevented its stable encapsulation. There was a positive correlation between micelle absorbance at 808 nm and maximum temperature changes, indicating that micelles with a strong absorption closer to the laser wavelength produce the most heat (Figure S3). CuNC(Octa), which exhibited the highest absorbance at 808 nm, also achieved the highest increase in temperature, 40°C within 10 minutes. This increase in temperature is well beyond the threshold necessary to induce cell death. In fact, at temperatures over 60°C (i.e., temperature increase of 23°C), cell death occurs rapidly due to protein denaturation and cell membrane disruption. In contrast, the water control did not surpass a 5°C increase in temperature in the same time frame. Irradiation of ICG initially led to rapid heating of the sample, but after just a few minutes, the temperature began to decline (Figure 2B). Repeated cycles of heating of ICG showed progressive thermal degradation, with each round reaching a lower peak temperature than the previous. The thermal instability of ICG has often been cited as a limitation in its use as a PTT agent 47, 48. In contrast, the PC/NC-loaded micelles retained their ability to generate heat through multiple rounds of irradiation. CuNC(Octa)-loaded micelles were also examined for their ability to produce reactive oxygen species (ROS), which was monitored using diphenylisobenzofuran (DFBF). Irradiated CuNC(Octa)-loaded micelles demonstrated little ROS generation (Figure S4).

### *In Vitro* Imaging of NC and PC Micelles

**Figure 3:**
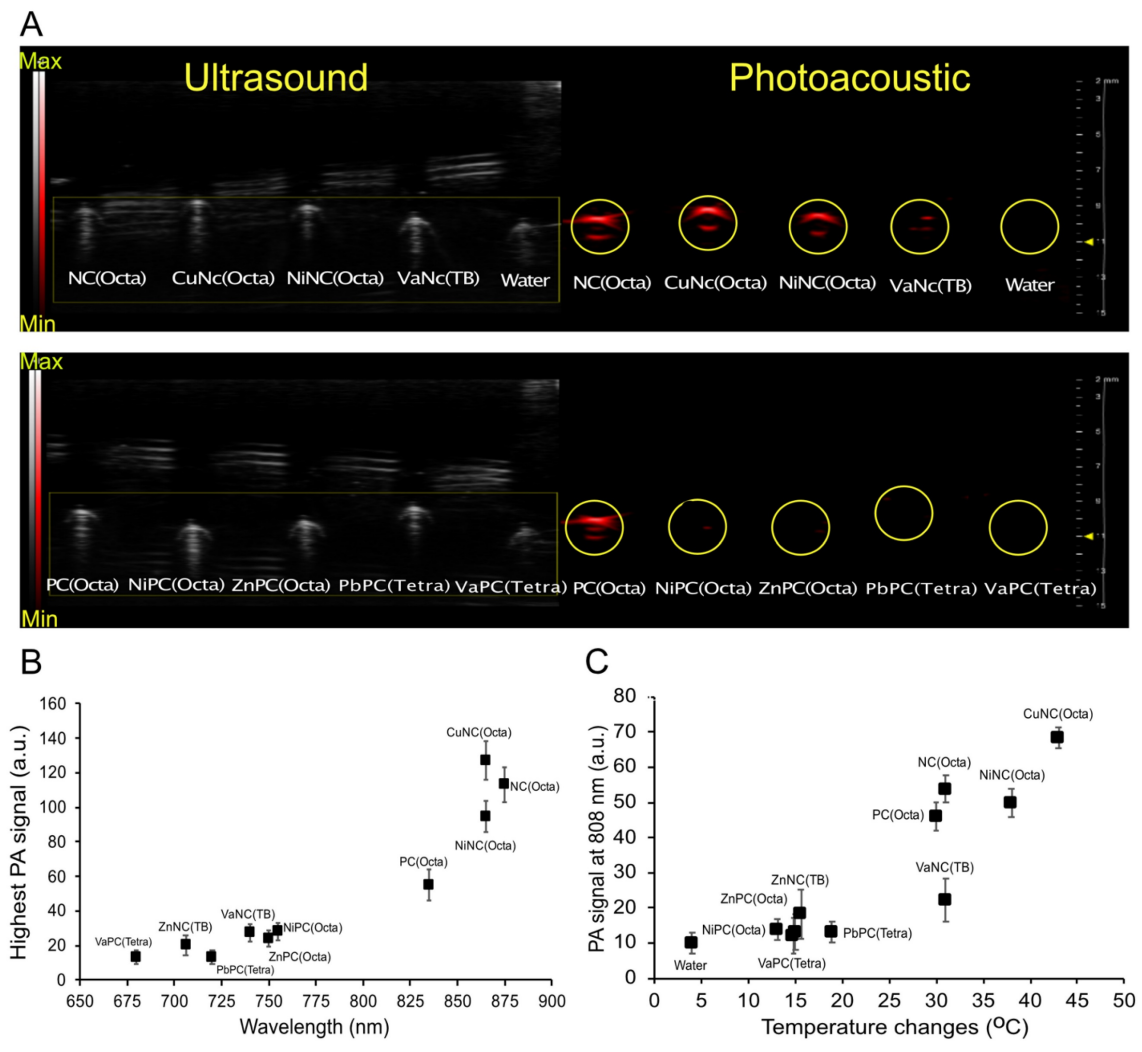
Ultrasound and photoacoustic phantom imaging (a cross-section view of the tubing). (A) The PA signal (denoted via a yellow circle) from NC-loaded micelles (top) and PC-loaded micelles (bottom). (B) The highest PA signal intensity of different NC/PC-loaded micelles at their highest PA wavelength in equivalent concentration. (C) The comparison between the PA signal of different NC/PC-loaded micelles versus temperature changes at 808 nm wavelength (note that the graph combines data from two separate experiments).

Aqueous solutions of select PC and NC micelles or water were loaded into imaging phantoms (Figure S5A), and the photoacoustic spectra were collected (Figure S5B). As expected, the photoacoustic spectra generally mirrored the absorbance spectra of the respective dyes. The micelles mostly displayed increased photoacoustic signal intensity compared to the control filled with water (Figure 3A, Figure S5C). After normalizing the data across imaging trials, CuNC(Octa) showed the highest PA signal (Figure 3B). A comparison of PA signal intensity at 808 nm vs temperature increase (i.e., from separate experiments) generated by the micelles revealed CuNC(Octa) to have the highest values across both variables, and the water control to have the lowest (Figure 3C).

### Cell Toxicity of NC and PC Micelles

**Figure 4:**
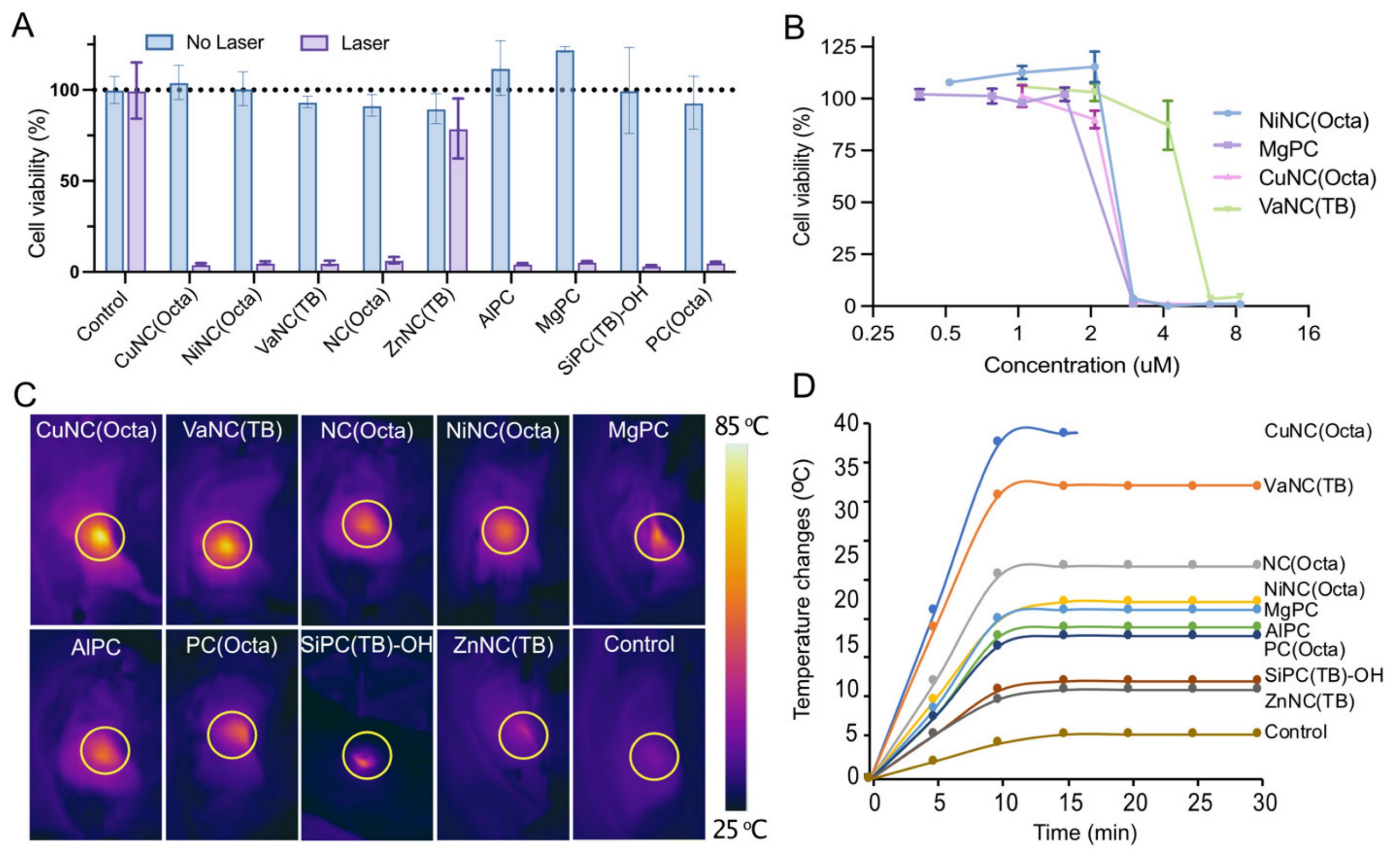
(A) Viability of breast cancer cells (4T1) incubated with different NC/PC-loaded micelles in darkness and under laser irradiation (808 nm, 07 W/cm^2^, 5 min). (B) Dose-response curves of 4T1 cells treated with different concentrations of the four best NC/PC-loaded micelles at 808 nm light for 5 min (0.7 W/cm^2^). (C) Infrared thermography and (D) temperature elevation of tumors treated with saline (control), and different NC/PC-loaded micelles (10 mg/kg) upon 30 minutes of laser irradiation. The yellow circle indicates the tumor site. The group of CuNC(Octa) mice experienced significant increases in temperature, which could lead to harm. To prevent further damage, the animal experiment was halted after 15 minutes.

The cytotoxicity was evaluated for the nine micelle formulations that exhibited the most heating upon irradiation with 808nm light. The micelles (25 μM) or fresh media were incubated with mouse 4T1 mammary carcinoma cells, an aggressive triple-negative breast cancer cell line, and then irradiated with an 808 nm laser (0.7 W/cm^2^, 5 minutes). Cellular viability was quantified by MTS assay. Treatment with micelles in combination with laser irradiation (0.7 W/cm^2^, 5 minutes) sharply decreased the cell viability of all groups to below 5%, except for the ZnNC(TB)-loaded micelles (Figure 4A). This effect was not observed without laser irradiation, suggesting that the micelles would be well-tolerated outside any irradiated areas. Dose-response experiments of the four best heating micelles revealed a sharp threshold between non-effective and cytotoxic doses (Figure 4B). A dose of 3.125 µM was sufficient to kill >95% of cancer cells when dosing with NiNC(Octa)-, MgPc-, and CuNC(Octa)-loaded micelles.

### *In Vivo* Heating of NC and PC Micelles

The *in vivo* efficacy of PA imaging and PTT can be influenced by the intratumoral accumulation and distribution of the PC/NC-loaded micelles, in addition to their photothermal conversion efficiency. Therefore, a preliminary screen of the nine selected micelle formulations was performed in a syngeneic 4T1 murine tumor model, whereby the PA signal and tumor temperature were compared upon irradiation with an 808nm laser. Mice with established 4T1 flank tumors were either dosed intravenously with 5 mg/kg of micelles or saline. After 24 hours, the tumors showed pigmentation, indicating the accumulation of the NC/PC micelles within the tumors. This was verified by PA imaging (Figure S6A and B). It has previously been reported that phthalocyanine nanoparticles tend to accumulate more in tumor sites and less in healthy organs after 24 hours 46. This helps to avoid damage to noncancerous cells/tissues under PTT treatment. A strong PA signal was observed within the tumor for all nine micelle formulations evaluated, but not saline. A comparison of the PA signal of different micelles at their respective peak wavelengths showed that the highest PA signal was observed in the mice dosed with CuNC(Octa) (Figure S6C).

Following PA imaging, the tumor area was irradiated with an 808 nm laser (0.7 W/cm^2^) for 30 minutes, and the temperature was monitored with a FLIR ONE thermal camera (Figure 4C). The tumors of all mice that received micelles increased to a higher temperature than control mice that received laser treatment but no micelles (Figure 4D). The skin temperature of irradiated tumors containing CuNC(Octa) reached 70°C (i.e., a temperature change of 40°C), the highest final temperature of the groups. The ablated tumor sites had scabbed the next day and subsequently healed over the next two weeks. Given the efficacy of CuNC(Octa) as a PA contrast agent and PTT agent both *in vitro* and *in vivo*, we elected to focus on this formulation for subsequent experiments.

To compare CuNC(Octa) to other photothermal therapy agent candidates, the heating efficacy of CuNC(Octa)-loaded micelles was compared with gold nanorods and gold nanospheres. At a concentration of 50 ug/ml, the heating efficacy of irradiated (0.7 W/cm^2^) CuNC(Octa)-loaded micelles was as good or better than an equivalent concentration of gold nanorods and nanospheres, respectively (Figure S7A). In addition, the PA signal from the CuNC(Octa)-loaded micelles was at least 10-fold greater in intensity than both gold particles (Figure S7B).

### *In Vivo* PTT to Treat a Murine Cancer Model

**Figure 5:**
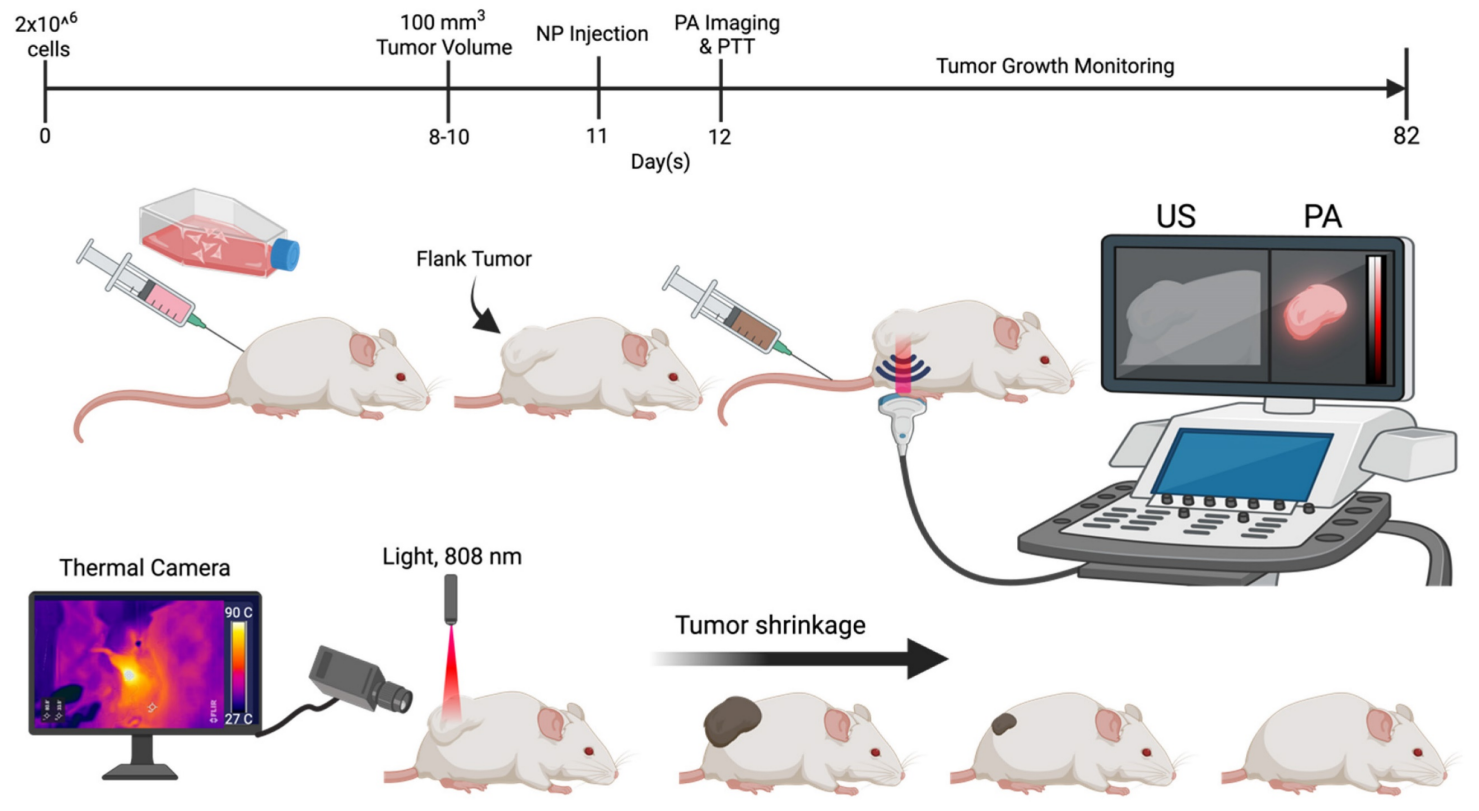
Animal study design. Schematic representation of 4T1 cell injection (flank tumor implantation), nanoparticle (NP) administration, PA imaging, and the timeline for mice treated with micelles.

**Figure 6:**
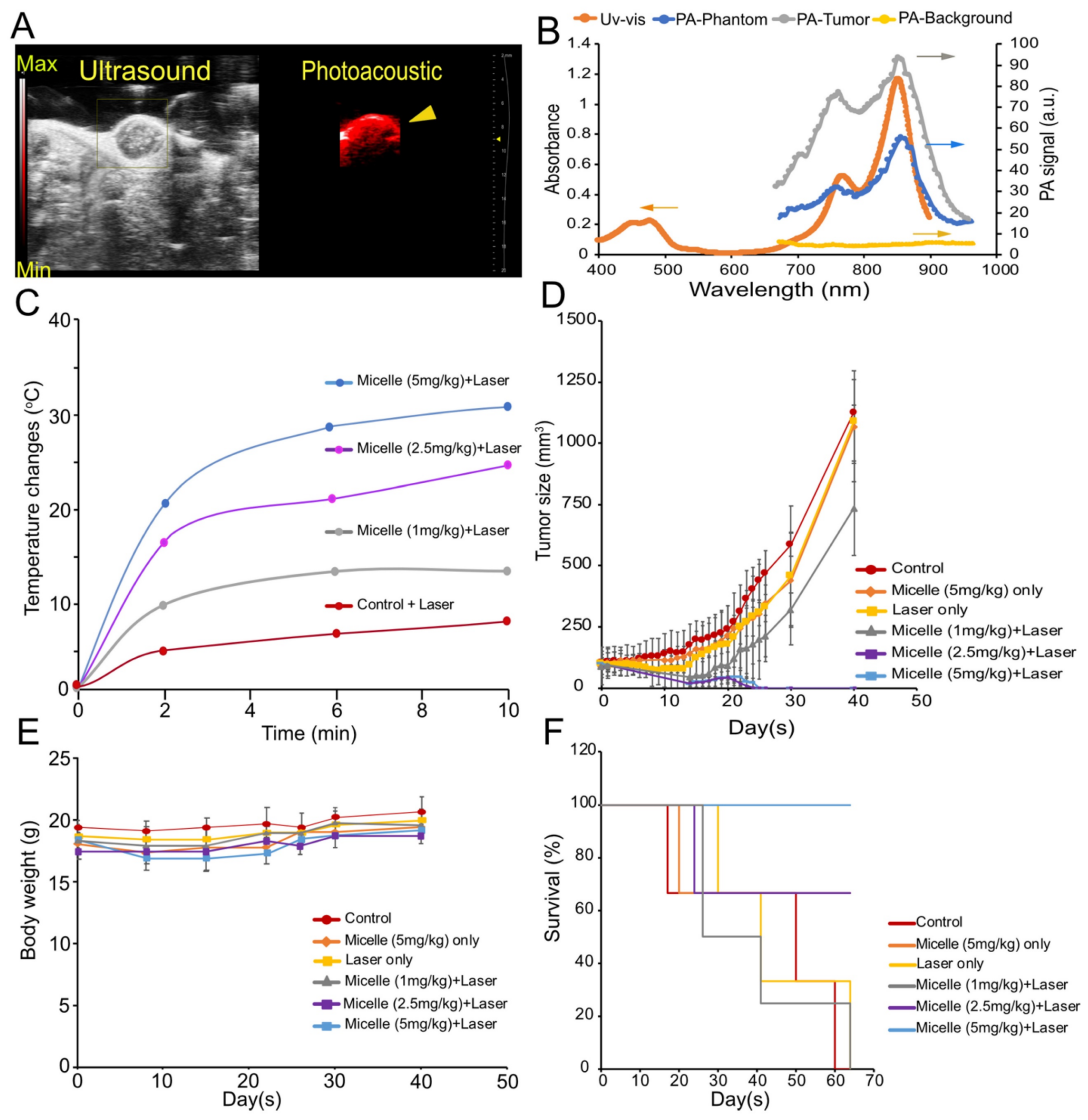
(A) Snapshot of the ultrasound and PA image of tumor-bearing mice following intravenous (retro-orbital) administration of 5 mg/kg CuNC(Octa) micelles at 870 nm excitation wavelength. (B) Uv-vis and the PA spectrum of CuNC(Octa)-loaded micelles. As shown, the spectrum from the Uv-vis and phantom align with the PA signal from the tumor. There is negligible PA signal from surrounding tumor tissues (PA-background) when PA gain is adjusted to 25 dB. (C) Temperature elevation of tumors treated (after 24 hours) with different injection doses (1, 2.5, and 5 mg/kg) of CuNC(Octa) micelles 10 minutes of laser irradiation. Measurements of tumor temperature acquired from thermographic images. (D) Tumor growth of mice treated with saline (control) and various concentrations of CuNC(Octa)(NP) with or without 10 minutes of laser irradiation (E) The body weight of 4T1 tumor-bearing mice was monitored after treatment. (F) Kaplan-Meier curves demonstrating animal survival for 70 days after photothermal treatment.

To further evaluate CuNC(Octa)-loaded micelles as a theranostic agent, we tested its efficacy at three different doses in mice bearing 4T1 tumors (Figure 5). Once the tumor volumes reached approximately 100 mm^3^, groups either received saline, 5 mg/kg CuNc(Octa)-loaded micelles only, laser treatment only, or CuNC(Octa)-loaded micelles (1 mg/kg, 2.5 mg/kg, or 5 mg/kg) and 808 nm laser irradiation, 24 hours post-intravenous administration. Just prior to PTT, micelle accumulation at the tumor was verified with PA imaging (Figure 6A). The PA spectrum within the tumor closely matched the absorbance and PA spectra of CuNC(Octa)-loaded micelles (Figure 6B), confirming that CuNC(Octa)-loaded micelles were present at the tumor site.

Due to the intensity of temperatures reached with the previous experiment, the PTT irradiation time was shortened to 10 minutes (0.7 W/cm^2^). Tumor heating with various injection doses of CuNC(Octa)-loaded micelles was monitored with an infrared camera and plotted over time (Figure 6C). Mice body weights and tumor volumes were monitored at intervals for 40 days following treatment. The mice that received saline, CuNC(Octa)-loaded micelles only, laser only, or 1 mg/kg CuNC(Octa)-loaded micelles + laser irradiation exhibited continual tumor growth after treatment (Figure 6D), although the group that received 1 mg/kg group did appear to have a slight delay in tumor growth. The groups that received 2.5 mg/kg and 5 mg/kg doses of CuNC(Octa)-loaded micelles + laser treatment showed no detectable tumors 25 days post-treatment. Body weight dipped briefly in mice that received 5 mg/kg of CuNC(Octa)-loaded micelles + laser treatment but recovered over time (Figure 6E), suggesting good treatment tolerability. All mice that received 5 mg/kg CuNC(Octa)-loaded micelles + laser treatment survived 70 days after treatment (Figure 6F). One mouse died from the 2.5 mg/kg group, which is suspected to be due to repeated exposure to anesthesia.

We present an in-depth screening of twenty-eight PC and nine NC dyes and derivatives formulated within micelles for potential dual PA imaging and photothermal therapy. Of the thirty-seven formulations, the CuNC(Octa)-loaded micelles were identified as the lead formulation due to its high heating efficiency and photoacoustic signal generation, both *in vitro* and *in vivo*. A dose as low as 2.5 mg/kg, followed by PTT, was sufficient to show complete tumor ablation in a murine model of triple-negative breast cancer, and all mice in the 5 mg/kg group survived. These results represent the first time CuNC(Octa) has been investigated as a PTT and PA imaging agent and offers an attractive alternative to ICG and gold nanoparticles due to its improved photostability and PA imaging capabilities, respectively.

Preparing oil-in-water emulsions with PEG-PCL as the stabilizing amphiphile provided a simple means to encapsulate the hydrophobic PC/NC dyes and allow for their solubilization in aqueous solvents. Encapsulation of the dye was robust, as noted by high encapsulation efficiency, and the micelles were found to be stable, as indicated by little to no change in the hydrodynamic diameters over 6 months. The simplicity of our method ensured facile reproducibility and repeatability (Table S3).

Although the encapsulation strategy allowed for the facile solubilization of NC and PC dyes and derivatives with FDA-approved materials, additional work may be needed for broad clinical applicability. As expected, the PDT effects of CuNC(Octa) was reduced when formulated within micelles, attributed to the reduced ability to form reactive oxygen species. Although not directly tested, this suggests reduced fluorescent properties of CuNC(Octa) when formulated within micelles, thereby likely limiting clinical fluorescent imaging applications. In this study, tumor localization and accumulation relied solely on the enhanced permeability and retention effect. An improvement in tumor specificity and possibly tumor accumulation could potentially be achieved with the addition of cancer-specific targeting ligands on the micelle surface. Further modulation of size and charge could also be explored to identify optimum formulations for tumor localization and accumulation. Lastly, we demonstrate proof-of-principle studies of PTT-mediated tumor ablation using CuNC(Octa)-micelles; however, other candidates from our initial *in vitro* screening appear promising, including PC(Octa), NC(Octa), and NiNC(Octa) (Figure 3), and could be explored.

## Conclusion

CuNC(Octa)-loaded micelles represent a promising theranostic agent for PA imaging and PTT. The ability to utilize conventional ultrasound in combination with PA imaging enables the simultaneous acquisition of information about tumor morphology and micelle accumulation. PTT with CuNC(Octa)-loaded micelles can lead to the complete eradication of highly invasive tumors. Although CuNc(Octa)-micelles were found to exhibit the highest signal by PA imaging and most efficient thermal conversion at 808 nm, compared to all of the other tested dyes, its peak absorbance is actually closer to 870 nm. This suggests that lasers emitting light at 870 nm could achieve an even further improvement in thermal conversion. In addition to further optimizing the micelles and laser irradiation for PA imaging and PTT, we also hope to utilize PA or magnetic resonance thermometry, to monitor the spatial temperature profile within living subjects, to ensure there are no detrimental effects caused by PTT owing to off-target thermal diffusion to surrounding normal tissues.

## Materials and Methods

### Materials

Poly (ethylene glycol)-b-poly(ε-caprolactone) (PEG4k-PCL3k) was purchased from Polymer Source (Quebec, Canada). Indocyanine green (ICG), tetrahydrofuran (THF), dimethyl sulfoxide (DMSO), dimethylformamide (DMF), and all PCs and NCs were purchased from Sigma-Aldrich (St. Louis, MO). Fetal bovine serum (FBS), Trypsin-EDTA, Dulbecco's Modified Eagle Medium (DMEM), and penicillin-streptomycin solution were purchased from Thermo Fisher Scientific (Gibco; Waltham, MA). The 3-(4,5-dimethylthiazol-2-yl)-5-(3-carboxymethoxyphenyl)-2-(4-sulfophenyl)-2H-tetrazolium (MTS) cell proliferation assay kit was purchased from Abcam. Pegylated Gold Nanoshells (AuNP, peak @ 800 nm, 160 nm diameter) and Gold Nanorods (AuNR, peak @ 800 nm, 55 nm x 15 nm) were obtained from nanoComposix.

### Preparation of NC and PC-Loaded Micelles

Various hydrophobic PC and NC derivatives were loaded into polymeric micelles, composed of the amphiphilic deblock copolymer poly (ethylene glycol)-(caprolactone) (PEG-PCL), via oil-in-water emulsions. A solution of PC/NC was created by dissolving 1.5 mg of dye in THF, DMF, or anhydrous DMSO. 80 μL of the PEG-PCL solution (50 mg of PEG-PCL in 1 mL of THF) was added into the PC/NC solution and vortexed. The resulting mixture was pipetted into a glass vial containing 4 mL of water, and the sample was sonicated (in a water bath sonicator) until a homogenous colloid was observed (5-10 min). To remove organic solvents, the emulsion was allowed to stand overnight to evaporate THF. DMF and DMSO samples were further purified through dialysis in pure water.

### Physiochemical Characterization

The micelles were characterized by UV-absorption spectroscopy in DMF, DMSO, THF, or water to capture their absorbance spectra (Varian-Cary 100 Bio spectrophotometer, Agilent Technologies, Santa Clara, CA). The hydrodynamic diameter and zeta potential of micelles were also examined using dynamic and electrophoretic light scattering, respectively (Zetasizer Nano ZS, Malvern Panalytical, Malvern, United Kingdom). Dye concentration was determined by dissolving the micelles in THF, DMSO, or DMF. The encapsulation efficiency and dye-loading were analyzed spectrometrically using the following equations:



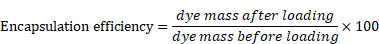





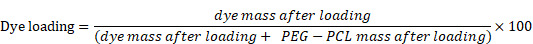



### *In Vitro* Heating

NC/PC-loaded micelle solutions in water and various controls were treated with laser irradiation to induce heating. 200 μL of micelle solutions at a concentration of 10 µM were added to a 96-well plate (clear with clear, flat bottom). A fiber optic thermometer (Nomad, Qualitrol-Neoptix, Fairport, NY) was inserted below the solution's surface. Solutions were irradiated with an 808 nm continuous wave (CW) laser (OEM Fiber Coupled Laser Systems, BWF5, B&WTek, DE, USA) at a power density of 0.7 W/cm^2^. Samples were irradiated for 10 minutes, and temperatures were recorded every 30 seconds. To evaluate photostability, samples were first irradiated for 10 minutes and then cooled to pre-irradiation temperature. Immediately after reaching this threshold, radiation was repeated for two additional 10-minute cycles with cooling in between. All experiments were performed at room temperature and with ambient light.

### *In Vitro* Imaging

Photoacoustic images were acquired using a Vevo LAZR system (VisualSonics Inc., Toronto, Canada) with excitation wavelengths from 680 nm to 970 nm, pulse duration 7-10ns, step size 1nm, frequency 20 Hz, and the 26mJ peak energy at the transducer end for all samples. The photoacoustic imaging signal was assessed by loading micelles at a concentration of 200 µM (based on the dye concentration) into polyethylene tubing (0.5 mm diameter). The tubes were placed in a plastic holder immersed in DI water. Images were acquired with the following settings: PA gains 40 dB, priority 99%, and distance 10 mm from the LZ250 transducer (75 µm axial resolution; 13-24 MHz broadband frequency).

### Cell Culture and Cell Viability by MTS Assay

4T1 murine breast cancer cells (ATCC, Rockville, MD) were cultured in DMEM supplemented with 10% heat-inactivated FBS and 1% penicillin-streptomycin (100 U mL-1 penicillin, 100 μg mL-1 streptomycin). Cultures were maintained at 37 °C in a humidified incubator with 5% CO_2_. The cytotoxicity of NC/PC-loaded micelles was tested using 4T1 breast cancer cells seeded in 96-well plates at a density of approximately 1.0 × 10^4^ per well. Cells were allowed to incubate overnight. Media was removed from individual wells, replaced with fresh media containing NC/PC-loaded micelles at various concentrations (n = 3 wells per condition), and incubated with the micelles for 24 hours. Select wells were irradiated with an 808 nm laser at 0.7 W/cm^2^ power for 10 minutes. 24 hours after irradiation, media was removed, and cells were washed with phosphate-buffered saline. Finally, fresh media was added to the wells and subjected to a standard MTS assay. Results were expressed as a percentage of cell viability compared to the average absorbance value (λ = 490) of cells treated with media alone (n = 3 wells per plate used).

### *In Vivo* Imaging

Female mice (BALB/c), aged approximately 6-8 weeks, were obtained from Charles River Laboratories (Wilmington, MA). All animal studies were approved by the University of Pennsylvania Institutional Animal Care and Use Committee, per AAALAC guidelines and accreditation. Mice were fed standard chow ad libitum unless otherwise noted. 4T1 tumor cells (2 x 10^6^ cells in 100 ul) were implanted in the right flank of BALB/c mice. When the tumor size reached approximately 100 mm^3^, mice were anesthetized with isoflurane, the mice hair was removed via depilatory cream, and pre- and post-imaged by PA imaging with the following settings: excitation wavelengths from 680 nm to 970 nm, pulse duration 7-10ns, step size 1nm, and frequency 20 Hz, PA gains 25 dB (to reduce the PA signal from the healthy and tumor tissues or any endogenous contrast), priority 99%, and distance 5 mm from the LZ250 transducer. Later the same day, mice were injected intravenously (I.V.) (retro-orbitally) with micelles at a dose of 5 mg/kg body weight (n = 3 mice per micelle). Control mice were not injected with micelles. The mice were post-imaged by PA imaging after 24 hours. Snapshots of images were represented at their maximum absorbance peak based on the PA spectrum.

### *In Vivo* Heating

All mice were subjected to subcutaneous photothermal therapy one day after PA imaging. Mice were anesthetized with isoflurane, and body temperature was maintained thereafter using a recirculating water pad (Kent Scientific, Torrington, CT). Tumors were irradiated at 808 nm (0.7 W/cm^2^, which fully covered all tumors). Tumor temperatures were monitored using a FLIR ONE thermal imaging camera (FLIR Systems, Wilsonville, OR) at the following time points: pre-treatment, 5, 10, 15, 20, 25, and 30 minutes. Images were later analyzed for maximum temperature in the tumor. Tumor volume was monitored and calculated as described below, starting with tumor cell implantation and continuing until animal death. Mice with tumor lengths surpassing 15 mm were sacrificed. Animals were also monitored for weight change.



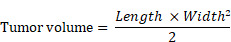



## Supplementary Material

Supplementary figures and tables.
