# Supplementary material for: Theranostic Phthalocyanine and Naphthalocyanine Nanoparticles for Photoacoustic Imaging and Photothermal Therapy of Tumors
Source: Nanotheranostics. 2024 Jan 1;8(1):100–11. doi: 10.7150/ntno.88892 (PMC10750118; doi:10.7150/ntno.88892)
Supplement: Supplementary file 1 — Supplementary figures and tables. [file ntnov08p0100s1.pdf]

Supporting Information

Theranostic Phthalocyanine and Naphthalocyanine Nanoparticles for Photoacoustic Imaging and Photothermal Therapy of Tumors

Yiran Tian, Nicole Carrillo-Malani, Kailin Feng, Joann Miller, Theresa M. Busch, Karthik M. Sundaram, Zhiliang Cheng, Ahmad Amirshaghaghi* and Andrew Tsourkas*

Yiran Tian, Nicole Carrillo-Malani, Kailin Feng, Dr. A. Amirshaghaghi, Prof. Z. Cheng, Prof. A. Tsourkas

Department of Bioengineering

University of Pennsylvania

Philadelphia, PA 19104, USA

*E-mail: atsourk@seas.upenn.edu

*E-mail: ahmadam@seas.upenn.edu

Prof. K. M. Sundaram

Department of Radiology,

Hospital of University of Pennsylvania,

Philadelphia, PA 19104, USA

Joann Miller, Prof. Theresa M. Busch

Department of Radiation Oncology,

Perelman School of Medicine, University of Pennsylvania,

Philadelphia, PA, 19104, USA

TABLES

Table S1. Size, PDI, and zeta potential of different NC/PC-loaded micelles.

Samples	Hydrodynamic Dimeter (nm)	PDI	Zeta Potential (mV)
CuNC(Octa)	64.85	0.16	-31.50
NiNC(Octa)	70.10	0.19	-35.07
NC(Octa)	62.62	0.15	-31.70
VaNC(TB)	81.03	0.17	-37.90
ZnNC(TB)	61.79	0.15	-32.67
PC(Octa)	60.87	0.33	-34.80
MgPC	63.60	0.28	-12.91
AlPC	36.51	0.51	-37.63
SiPC-(TB)-OH	90.51	0.35	-0.70
ZnPC(TB)	55.32	0.18	4.25
Empty micelle	35.41	0.23	-25.17

Table S2. CuNC(Octa)-loaded micelle stability. Particle size based on DLS mean intensity (%) measurements and PDI taken over a total of 10 days in 10% fetal bovine serum (FBS).

Day(s)	Hydrodynamic Dimeter (nm)	PDI
0	69.82	0.278
1	71.20	0.27
2	70.53	0.27
3	69.22	0.271
4	73.20	0.27
5	72.98	0.265
6	73.81	0.259
7	74.02	0.260
8	71.53	0.220
9	70.76	0.285
10	72.34	0.275

Table S3. Repeatability of CuNC(Octa)-loaded micelle synthesis (ratio 1:2.7 w/w of dye/polymer)

	Batch 1	Batch 2	Batch 3	Average	St. Dev
Hydrodynamic Diameter (nm)	66.26	64.85	69.65	66.92	2.46
PDI	0.221	0.162	0.196	0.193	0.030
Encapsulation Efficiency (%)	96	95	98	96	1.53
Dye Loading (%)	26.2	25.9	26.7	26.2	0.40
Zeta-Potential (mV)	-28.00	-31.50	-26.00	-28.50	2.80

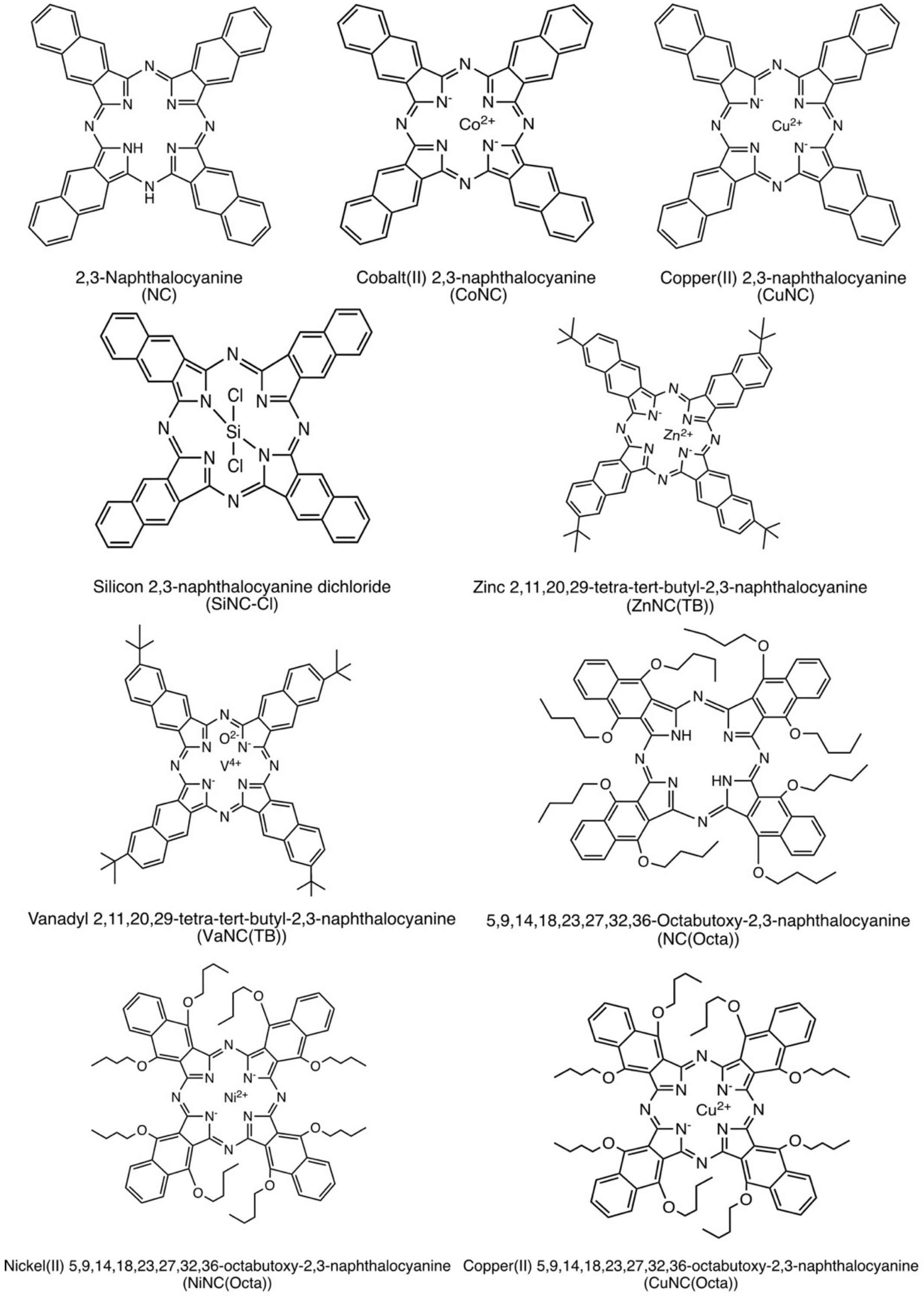

Figure S1. Chemical structures of the naphthalocyanine (NC) and phthalocyanine (PC) dyes used in this study.

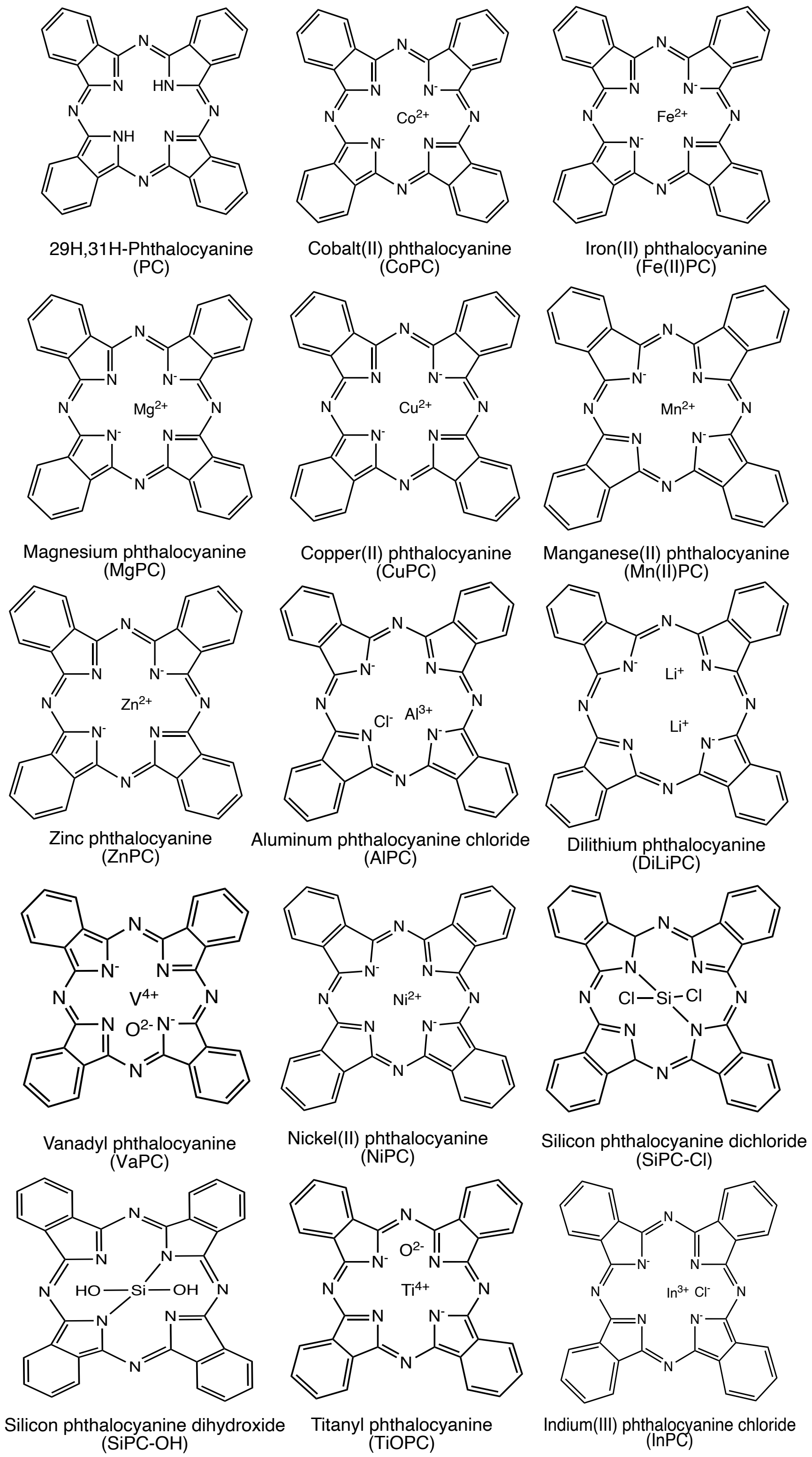

Figure S1 (continued). Chemical structures of the naphthalocyanine (NC) and phthalocyanine (PC) dyes used in this study.

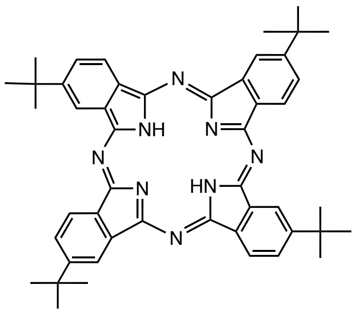

2,9,16,23-Tetra-tert-butyl-29H,31H-phthalocyanine
(PC(TB))

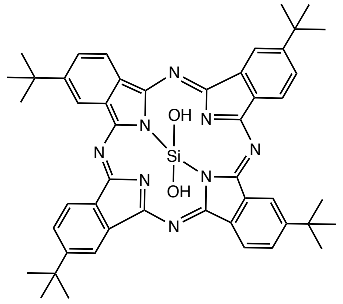

Silicon 2,9,16,23-tetra-tert-butyl-29H,31H-phthalocyanine dihydroxide
(SiPC(TB)-OH)

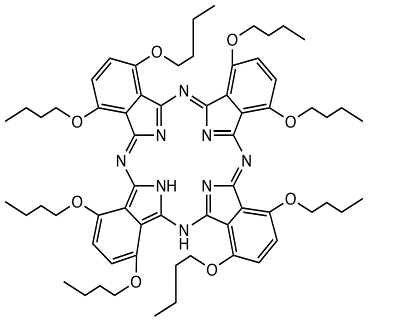

1,4,8,11,15,18,22,25-Octabutoxy-29H,31H-phthalocyanine
(PC(Octa))

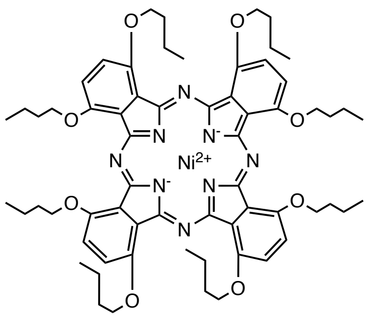

Nickel(II) 1,4,8,11,15,18,22,25-octabutoxy-29H,31H-phthalocyanine
(NiPC(Octa))

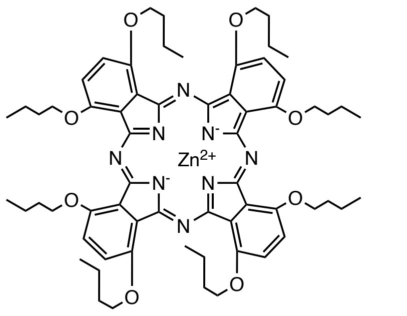

Zinc 1,4,8,11,15,18,22,25-octabutoxy-29H,31H-phthalocyanine
(ZnPC(Octa))

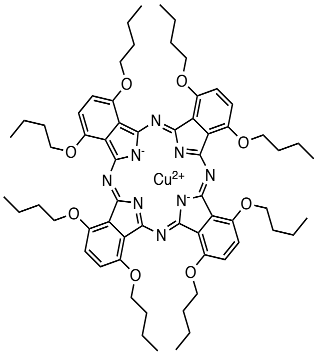

Copper(II) 1,4,8,11,15,18,22,25-octabutoxy-29H,31H-phthalocyanine
(CuPC(Octa))

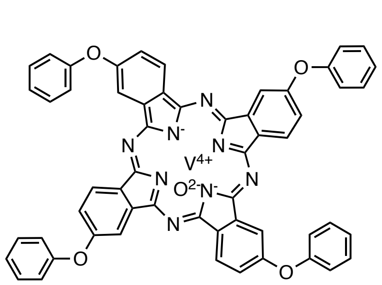

Vanadyl 2,9,16,23-tetraphenoxy-29H,31H-phthalocyanine
(VaPC(Tetra))

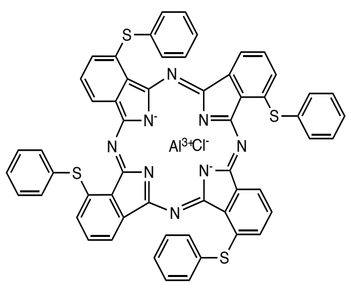

Aluminum 1,8,15,22-tetrakis(phenylthio)-29H,31H-phthalocyanine chloride
(AlPC(Tetra))

Figure S1 (continued). Chemical structures of the naphthalocyanine (NC) and phthalocyanine (PC) dyes used in this study.

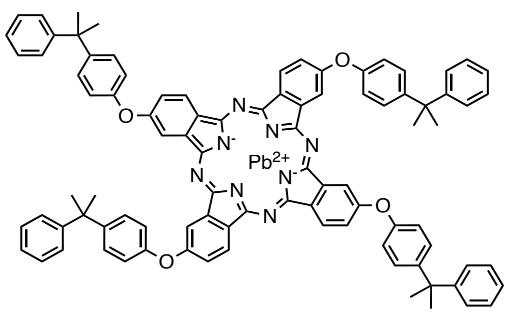

Lead(II) tetrakis(4-cumylphenoxy)phthalocyanine
(PbPC(Tetra))

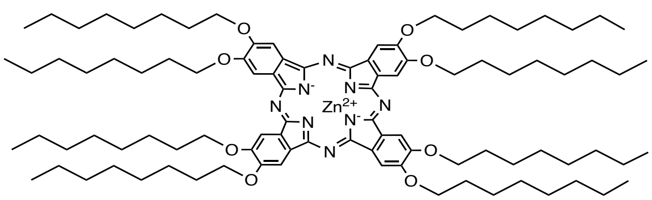

Zinc 2,3,9,10,16,17,23,24-octakis(octyloxy)
-29H,31H-phthalocyanine (ZnPC(Octakis))

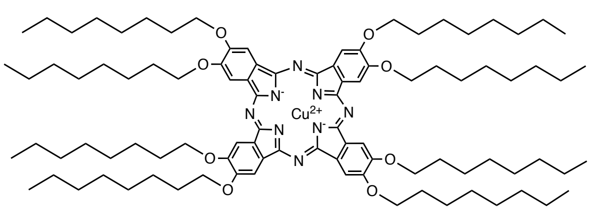

Copper(II) 2,3,9,10,16,17,23,24-octakis(octyloxy)
-29H,31H-phthalocyanine (CuPC(Octakis))

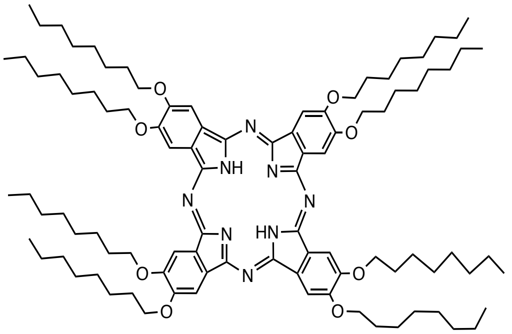

2,3,9,10,16,17,23,24-Octakis(octyloxy)-29H,31H-phthalocyanine
(PC (Octakis))

Figure S1 (continued). Chemical structures of the naphthalocyanine (NC) and phthalocyanine (PC) dyes used in this study.

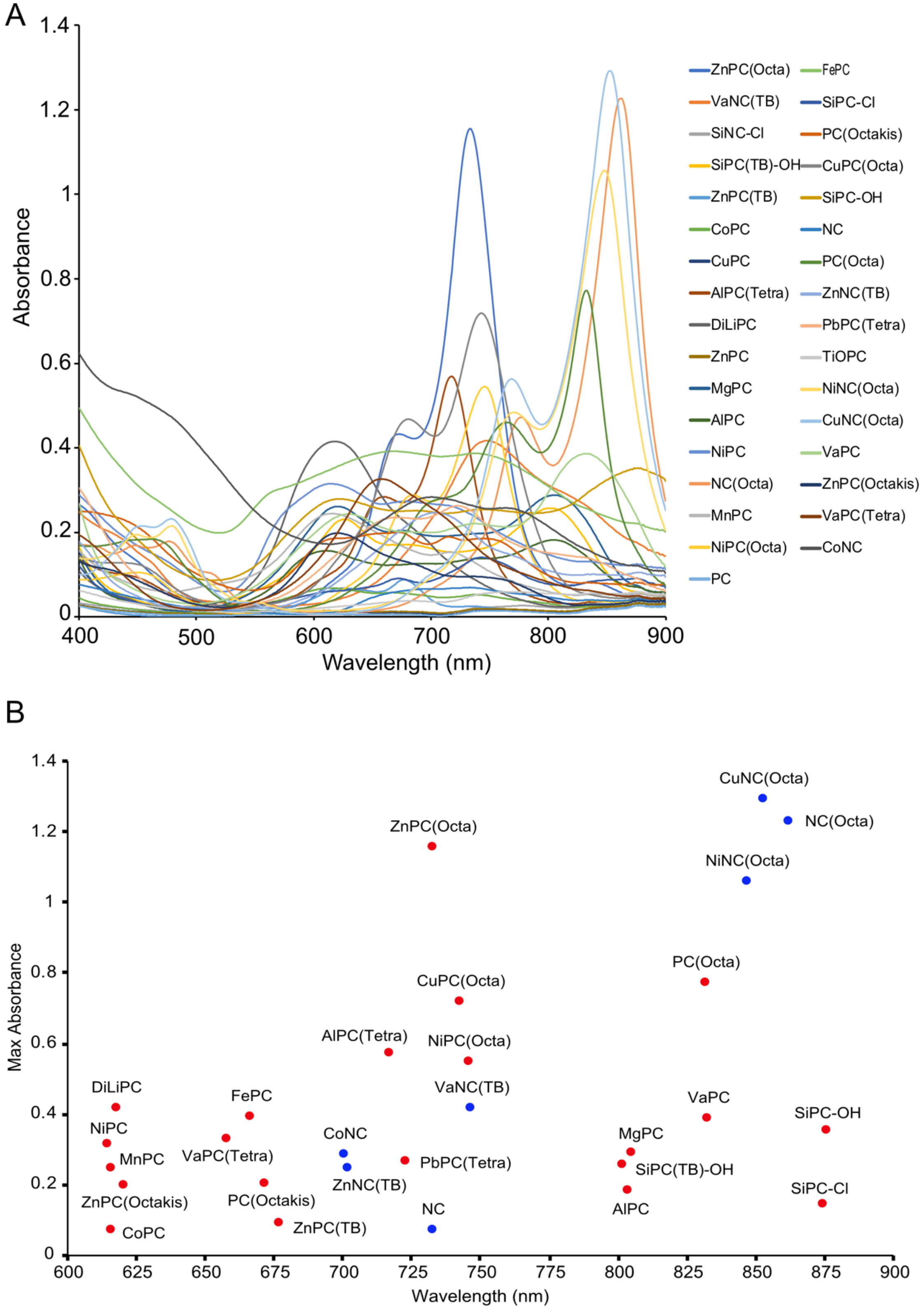

Figure S2. (A) Absorbance spectra of all NC/PC-loaded micelles. (B) The peak absorbance of each NC/PC-loaded micelles versus the corresponding peak wavelength. The blue and red dots represent NC and PC-loaded micelles, respectively. All measurements were made at an equivalent dye concentration (10 μM).

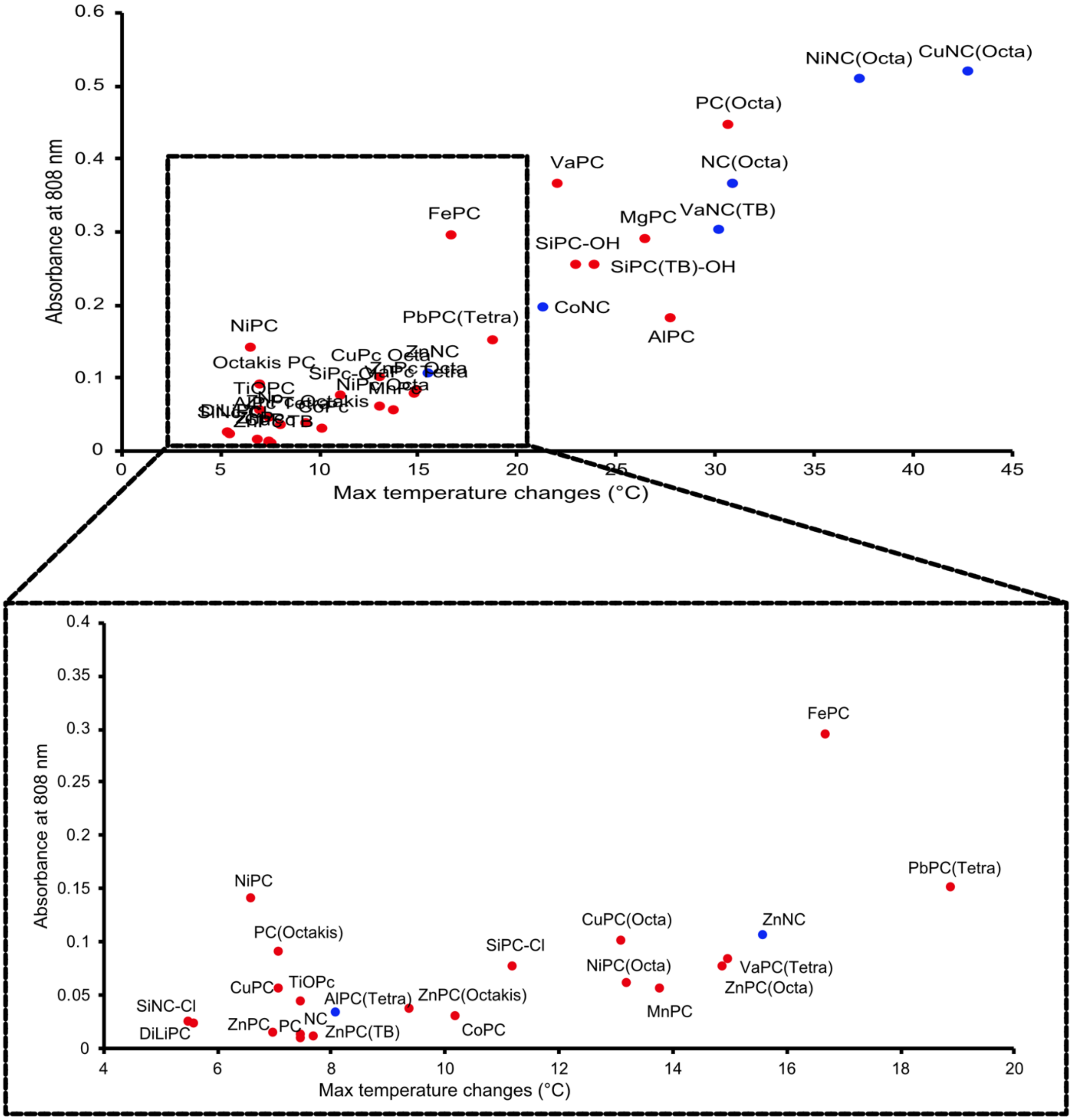

Figure S3. The absorbance of all NC/PC-loaded micelles at 808 nm versus maximum temperature changes. A dye concentration of 10 μM was used for all studies. Heating experiments were performed by irradiating samples with an 808 nm laser (0.7 W/cm^2) and measuring the temperature after 10 minutes.

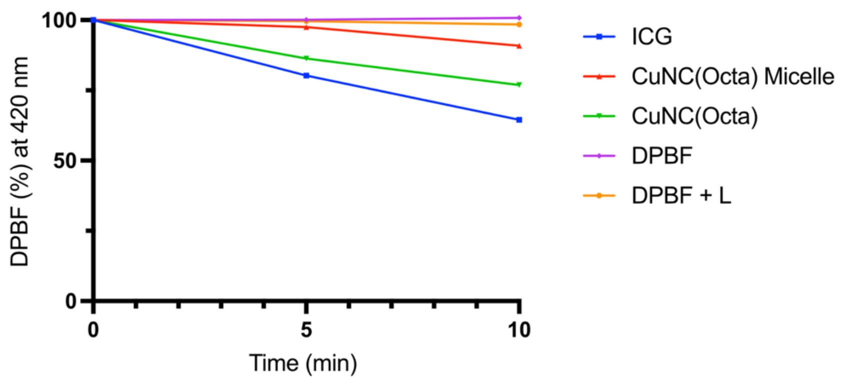

Figure S4. CuNC(Octa)-loaded micelles, free CuNC(Octa) in a mixture of water/DMSO (to improve the solubility of free CuNC(Octa)), and ICG at equivalent dye concentrations (10 μM) were irradiated (808 nm, 0.7 W/cm^2) in the presence of DPBF, which detects the formation of reactive oxygen species. DPBF alone was also measured in the presence and absence of laser irradiation (L) to demonstrate the stability of the probe during the experiment.

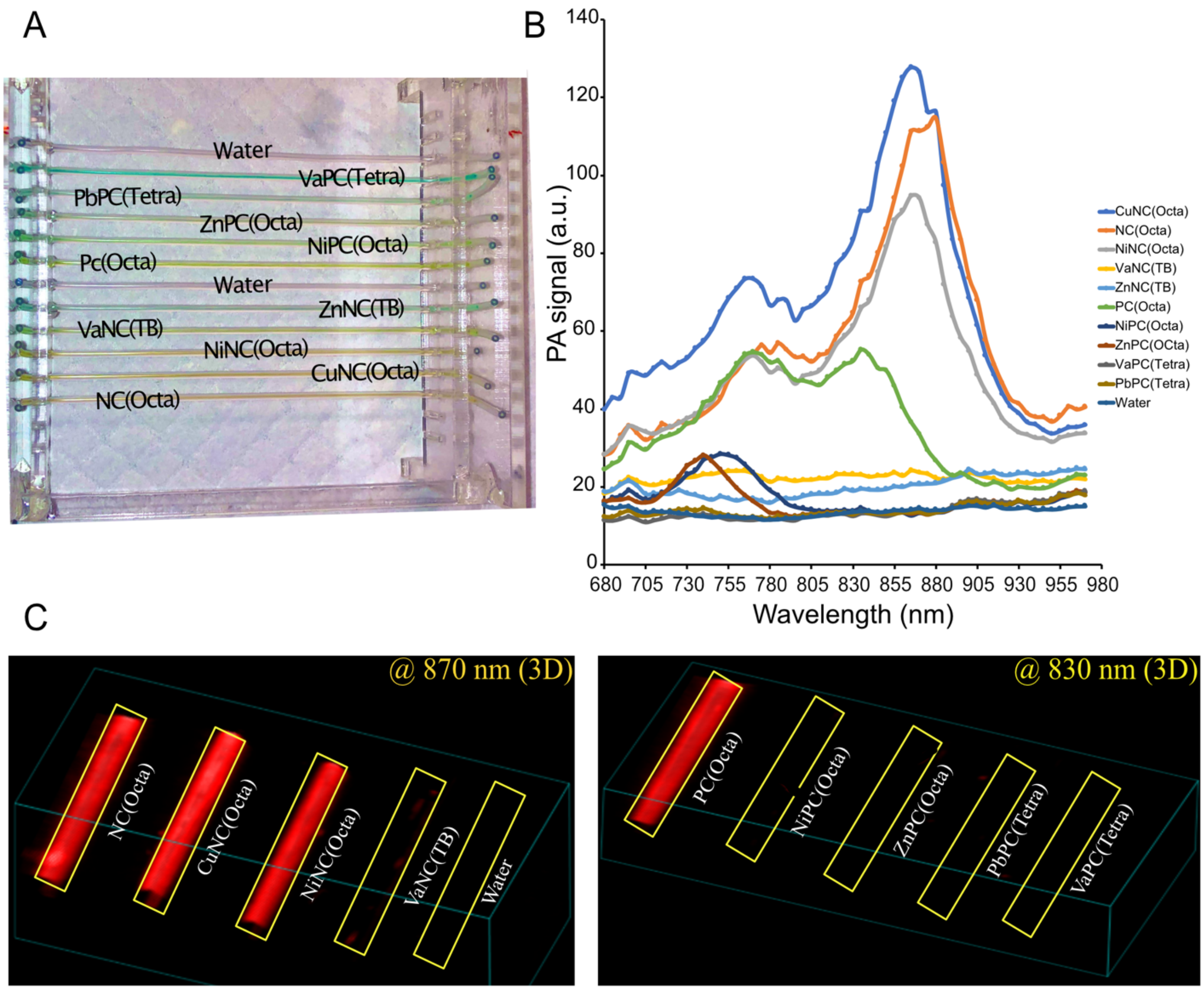

Figure S5. (A) Home-made phantom imaging chamber with flexible plastic tubing (10-15 cm) filled with select PA contrast agents or degassed water. (B) The PA spectrum (from 680-970nm) of select contrast agents. (C) Three-dimensional (3D) PA images of the phantom, with NC and PC-loaded micelles, scanned using an 870 nm and 830 nm excitation wavelength.

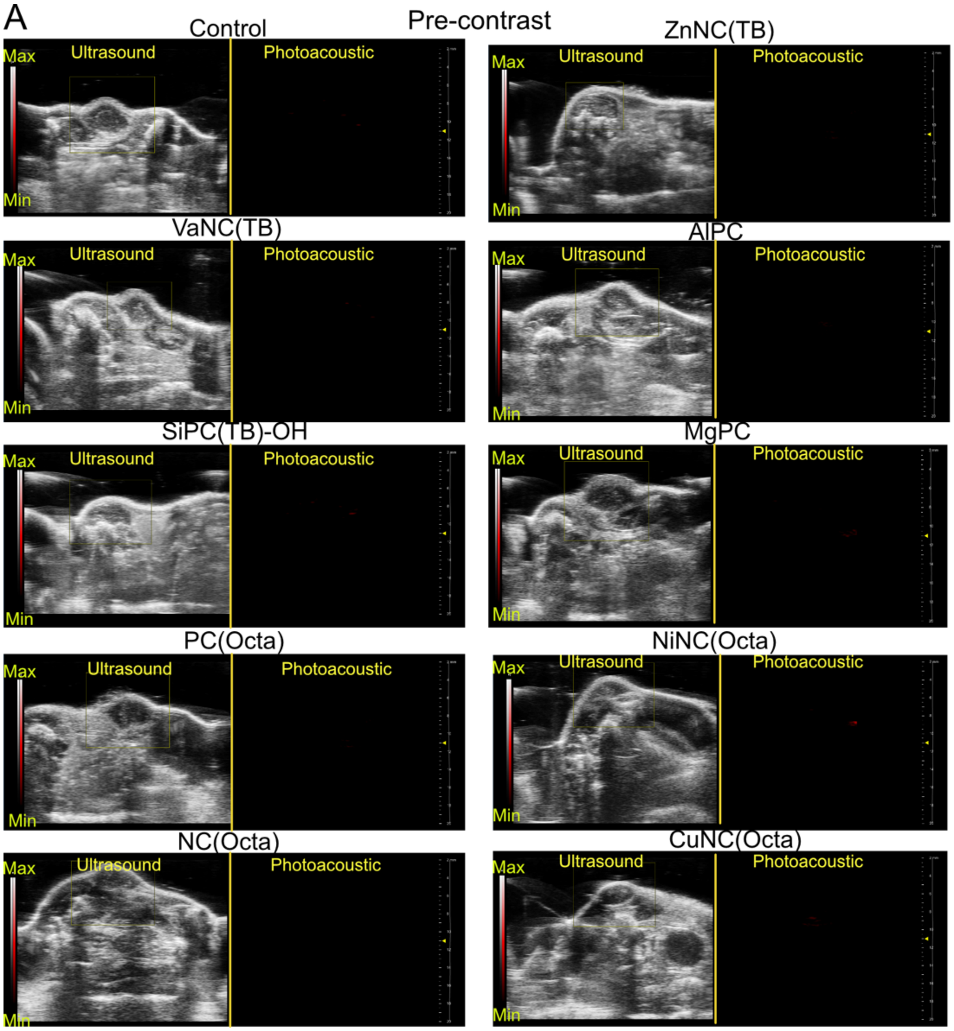

Figure S6. PA images of 4T1 tumors (A) before intravenous injection of different NC/PC-loaded micelle. Images shown are at the highest absorbance peak wavelength of the respective micelle formulation. Images were acquired using excitation wavelengths ranging from 680 nm to 970 nm. The images were captured at an adjusted PA gain of 25 dB.

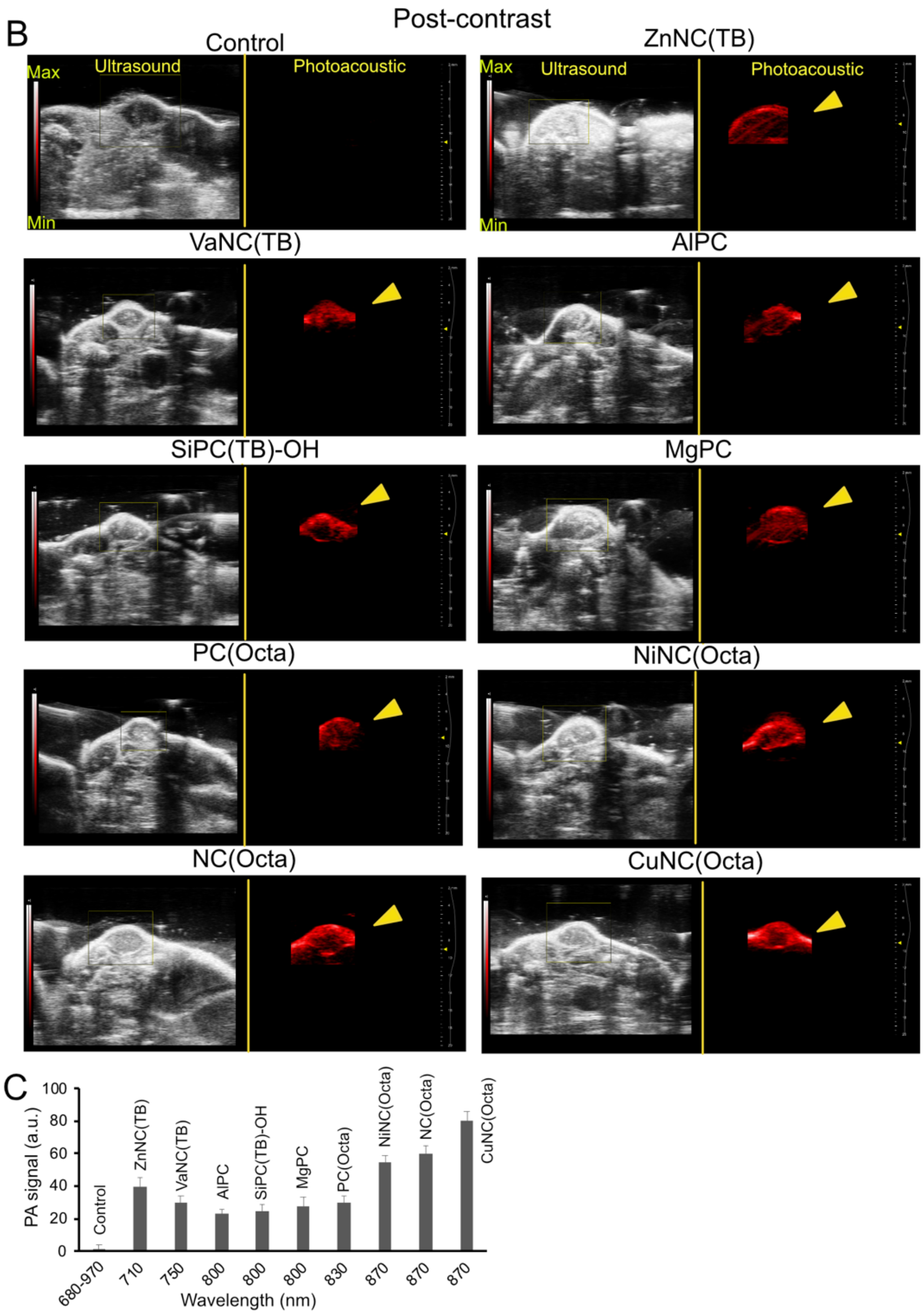

Figure S6 (continued). PA images of 4T1 tumors (B) 24 hrs after intravenous injection of different NC/PC-loaded micelle. Images shown are at the absorbance peak wavelength of the respective micelle formulation. Images were acquired using excitation wavelengths ranging from 680 nm to 970 nm. The images were captured at an adjusted PA gain of 25 dB. (C) Quantification of the PA signal of different NC/PC-loaded micelles at their respective peak absorbance wavelength. An equivalent dose of dye (5 mg/kg) was used for all injections.

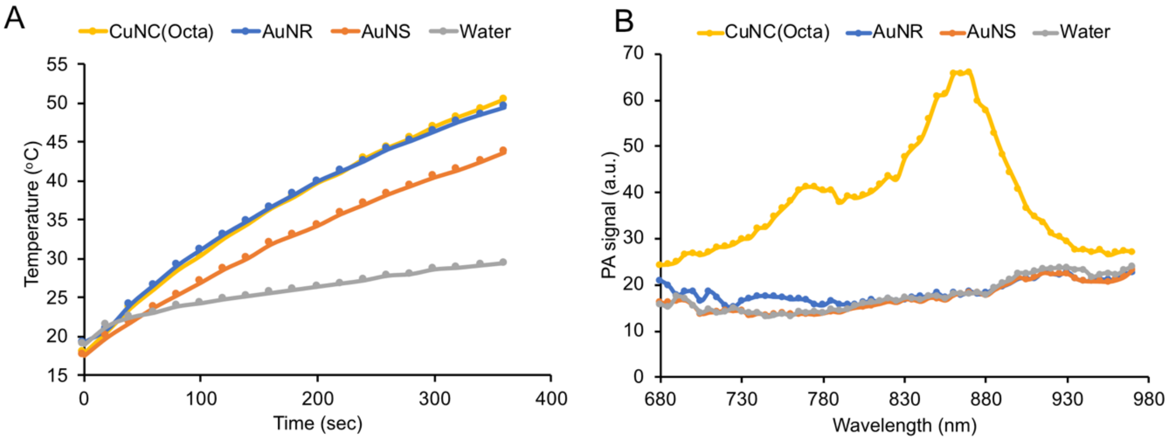

Figure S7. (A) Heating (808nm, 0.7 W/cm^2) and (B) the PA spectrum of samples containing CuNC(Octa) micelles, gold nanorods (AuNR), or gold nanoshells (AuNS). All measurements were acquired at equivalent concentrations for CuNc(Octa) micelles, gold nanorods, and gold nanoshells (50 μg/ml).
